# Benchmarking DNA large language models on quadruplexes

**DOI:** 10.1016/j.csbj.2025.03.007

**Published:** 2025-03-07

**Authors:** Oleksandr Cherednichenko, Alan Herbert, Maria Poptsova

**Affiliations:** a International Laboratory of Bioinformatics, HSE University, Moscow, Russia; b InsideOutBio, Charlestown, MA, USA

**Keywords:** Foundation model, Large language model, DNABERT, HyenaDNA, MAMBA-DNA, Caduseus, Flipons, Non-B DNA, G-quadruplexes

## Abstract

Large language models (LLMs) in genomics have successfully predicted various functional genomic elements. While their performance is typically evaluated using genomic benchmark datasets, it remains unclear which LLM is best suited for specific downstream tasks, particularly for generating whole-genome annotations. Current LLMs in genomics fall into three main categories: transformer-based models, long convolution-based models, and state-space models (SSMs). In this study, we benchmarked three different types of LLM architectures for generating whole-genome maps of G-quadruplexes (GQ), a type of flipons, or non-B DNA structures, characterized by distinctive patterns and functional roles in diverse regulatory contexts. Although GQ forms from folding guanosine residues into tetrads, the computational task is challenging as the bases involved may be on different strands, separated by a large number of nucleotides, or made from RNA rather than DNA. All LLMs performed comparably well, with DNABERT-2 and HyenaDNA achieving superior results based on F1 and MCC. Analysis of whole-genome annotations revealed that HyenaDNA recovered more quadruplexes in distal enhancers and intronic regions. The models were better suited to detecting large GQ arrays that likely contribute to the nuclear condensates involved in gene transcription and chromosomal scaffolds. HyenaDNA and Caduceus formed a separate grouping in the generated de novo quadruplexes, while transformer-based models clustered together. Overall, our findings suggest that different types of LLMs complement each other. Genomic architectures with varying context lengths can detect distinct functional regulatory elements, underscoring the importance of selecting the appropriate model based on the specific genomic task.

The code and data underlying this article are available at https://github.com/powidla/G4s-FMs

## Graphical Abstract

**Figure:**
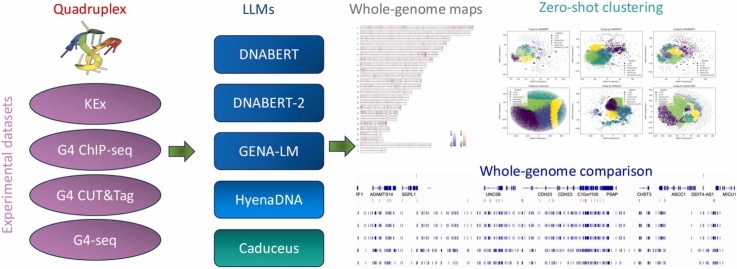

## 1 Introduction

Deep learning models for genomics have proven successful in predicting various functional genomic elements. Initially dominated by CNN and RNN architectures, these models have since evolved to transformers and, more recently, utilized capabilities of large language models. Large Language Models (LLMs), also known as foundation models (FMs), are pretrained on massive datasets and subsequently fine-tuned for specific downstream tasks, representing a powerful paradigm in modern machine learning. There are currently three types of LLMs trained on human genome: transformer-based (DNABERT [1], GENA-LM [2], Nucleotide Transformer [3]), CNN-based with long convolutions (HyenaDNA [4]), and RNN-like state-space models (SSMs) (Caduceus based on bidirectional MAMBA-DNA [5]).

Transformer [6] and its core attention layer [7] are widely used in foundation models. Self-attention is highly effective due to its capability to take into account all information within a context window, enabling it to model complex data relationships. Foundation models based on transformers in genomics have multiple successful implementations: DNABERT [1] and DNABERT-2 [8]; Nucleotide Transformer [3], and GENA-LM [2]. However, the key characteristic of transformers – attention – introduces fundamental limitations: the inability to model information outside context window and quadratic scaling with the window length. A vast body of research has emerged on more efficient variants of attention to address these limitations [9], often sacrificing the core properties that make attention so effective.

To address the challenges associated with transformers, Poli et al. [10] introduced Hyena, a model leveraging long convolutions and data-controlled gates. The adaptation of this model for long DNA sequence data is presented as HyenaDNA [4]. The method uses 1000 times fewer parameters (when compared to Nucleotide Transformer with billions of parameters) and is trained on 3000 times less data. It outperforms transformer approaches in time and memory usage.

Another alternative to transformer are SSMs, which have an entirely different RNN-like architecture. Gu et al. [11] introduced Mamba, an end-to-end neural network architecture that operates without attention blocks. Mamba was applied to DNA and directly compared to HyenaDNA under the same experimental setup. The most recent application of SSMs in genomics is Caduceus [5], which consists of two MAMBA DNA blocks with one trained on direct DNA sequence and the other on the reverse compliment.

Although all the aforementioned models demonstrated comparable performance on various genomic benchmark datasets, including promoters, enhancers, histone marks, and other regulatory elements, it remains unclear, which model is best suited for a specific genomic task. This is particularly challenging for the task of generating whole-genome predictions using a deep learning model trained on a limited dataset.

In this study, we aimed to address this issue by evaluating LLM models on quadruplexes – a class of flipons [12] that forms an alternative structure under physiological conditions rather than the canonical right-handed DNA and RNA helices. G-quadruplexes (GQs) present a unique class of genomic functional elements that have a predetermined regular sequence pattern in which four guanosines basepair to form tetrads that stack on each other to form a helix. By switching conformations, G-flipons can perform different functions depending on the position in the genome and the local context. The biological functions of GQ have been extensively studied (see [13] for a review). In promoters, GQ can act as activators or suppressors of transcription. When located in enhancers, they facilitate enhancer-promoter interactions, and play a critical role in chromatin organization, particularly at TAD boundaries and CTCF-binding sites. Additional functionality of GQ include involvement in DNA methylation, DNA repair, telomere maintenance, immunoglobulin gene rearrangement and other processes.

Whole-genome wet-lab experiments to detect GQs in DNA typically capture only a subset of structures present at the time of experiment and cannot provide a comprehensive whole-genome set of all functional elements. GQ have been investigated using various whole-genome experimental approaches, including ChIP-seq [14], CUT&Tag [15], G4-seq [16], permanganate nuclease footprinting (KEx) [17], and KAS-seq [18]. Reflecting the dynamical property of flipon formation, different experiments produce different GQ datasets confirming the need to reconstruct the reliable whole-genome map. Interpretation of such experiments is challenging as GQ can be formed by guanosines that are widely separated, giving rise to long loops between each strand of the GQ, or from different DNA strands, or from long GQ clusters [19]. The flip also may be transcription and cell-dependent and involve contributions of both RNA and DNA strands to the GQ.

Here we benchmarked three different types of large language models (LLMs) on all four major experimental quadruplex datasets, and then evaluated whole-genome predictions produced by models trained on each experimental dataset. This way we could assess not only performance of models on test sets but detect differences in LLM learning by analyzing de novo generated sets of GQs and the length of GQ containing segments found.

## 2 Materials and methods

### 2.1 Data preparation

We used four experimental datasets for quadruplex detection: permanganate/S1 nuclease footprinting method – KEx dataset [17] containing around 50 000 quadruplexes; G4 ChIP-seq dataset [14] containing about 9 000 quadruplexes; G4-seq dataset [16] containing almost 700 000 quadruplexes, G4 CUT&Tag [15] containing about 19 000 quadruplexes (Table 1).

**Table 1:** Experimental datasets for G-quadruplexes.

Dataset	Size	Mean length
G4 ChIP	8955	230
G4-seq	691503	252
G4 CUT&Tag	18288	127
KEx	53293	62

For negative labels we used randomly selected regions not overlapping with GQs. The resulting dataset had balanced classes with equal size of negative and positive samples. In addition to this task, we also addressed the fine-tuning on whole genome referring to [20].

### 2.2 Tokenization for G4s

Tokenization is a crucial aspect of language modeling, and for DNA language models, several techniques have been employed: k-mer tokenization [1], [3], base pair encoding [2], [21], and single nucleotide tokenization [4]. Different LLMs use different tokenization and we followed the author's guidance while using each model.

Researchers employing base pair encoding (BPE) often highlight the limitations of k-mer tokenization. For example, BPE enables the encoding of longer sequences using a smaller vocabulary [2]. Quadruplexes are relatively short sequences, and for this reason we utilized k-mers. This approach is more practical, as the occurrence of fine-tuned k-mers reflects characteristic GQ patterns, including flanks and regular or extended motifs [22].

### 2.3 Metrics of evaluation

To evaluate the performance of GQ detection we used standard metrics for classification task: accuracy, ROC-AUC, F1 score and Matthews correlation coefficient (MCC). We report average metrics calculated for cross-validation with 5 folds and present the results as mean ± standard deviation.

### 2.4 Fine-tuning

We followed standard procedures for fine-tuning LLM models. The training was conducted using A100, T4, and L4 GPUs. To address the issue of limited batch size, we employed gradient accumulation with a value of 8 using the Accelerate library. Training was performed over a small number of epochs, ranging from 4 to 10, depending on the model's performance. For larger models, such as DNABERTs and GENA-LM, performance was relatively high even in zero-shot classification, requiring fewer epochs to achieve optimal results. In contrast, Hyena-DNA and Caduceus required more epochs to reach comparable performance.

### 2.5 Low Rank Adaptation (LoRA)

To save time and memory during training, we utilized Low-Rank Adaptation (LoRA) [23]. This approach enables the fine-tuning of only a subset of model parameters, significantly reducing computational requirements without substantial loss in performance. The results of LoRA training for DNABERT, DNABERT 2 and GENA-LM are presented in Supplementary Tables 1–5.

## 3 Results

### 3.1 LLM comparison on quadruplex datasets

In this work we tested the following models and compare their performance on a classification task of quadruplex prediction. For transformer models, we fine-tuned pretrained DNABERT, DNABERT-2, and GENA-LM using various available numbers of parameters. We did not include Nucleotide Transformer in our benchmark study due to the limitations in computational resources. We fine-tuned HyenaDNA and Caduceus using 160 kB context and followed guidelines presented in the original papers. The models were chosen with the following number of total parameters: DNABERT (88 M) – large transformer with pretrained weights, DNABERT-2 (117 M) – large transformer with pretrained weights, GENA-LM (110 M) – large transformer with pretrained weights, Hyena-DNA (7 M) – small model based on long convolution trained from scratch, Caduceus (8 M) - small Mamba like model with pretrained weights.

W used four experimental quadruplex datasets – ChIP-seq [14], G4-seq [24], G4 CUT&Tag [15], KEx [17] (Table 1). The results of benchmarking are presented in Table 2, Table 3, Table 4, Table 5. Performance was evaluated using the Accuracy, ROC-AUC, F1 score and Matthew’s correlation coefficient (MCC). The ROC-AUC and accuracy metrics are high across all models, making it difficult to identify the superior model for the task. In contrast, the F1 score and Matthews Correlation Coefficient (MCC) are less biased and provide a more accurate assessment of model performance. Based on MCC and F1 scores, DNABERT2 and HyenaDNA demonstrate the best performance (Table 2, Table 3, Table 4, Table 5).

**Table 2:** LLM benchmark on G4 ChIP.

Model (#params)	Type	Accuracy	AUC	F1	MCC
DNABERT (88 M)	pretrained	95.0 ± 0.5	97.0 ± 0.5	85.0 ± 0.3	71.0 ± 0.3
**DNABERT−2 (117 M)**	pretrained	99.0 ± 0.5	99.0 ± 0.5	**89.0 ± 0.3**	**79.0 ± 0.3**
GENA-LM (110 M)	pretrained	98.0 ± 0.5	96.0 ± 0.5	88.0 ± 0.3	76.0 ± 0.3
HyenaDNA (7 M)	from scratch	97.0 ± 0.5	98.0 ± 0.5	83.0 ± 0.3	74.0 ± 0.3
Caduceus (8 M)	pretrained	96.0 ± 0.5	99.0 ± 0.5	86.0 ± 0.3	73.0 ± 0.3

**Table 3:** LLM benchmark on G4-seq.

Model (#params)	Type	Accuracy	AUC	F1	MCC
DNABERT (88 M)	pretrained	80.0 ± 0.5	95.0 ± 0.5	80.0 ± 0.3	66.0 ± 0.3
**DNABERT−2 (117 M)**	pretrained	91.0 ± 0.5	99.0 ± 0.5	**91.0 ± 0.3**	83.0 ± 0.3
GENA-LM (110 M)	pretrained	85.0 ± 0.5	94.0 ± 0.5	82.0 ± 0.3	73.0 ± 0.3
**HyenaDNA (7 M)**	from scratch	86.0 ± 0.1	98.0 ± 0.1	90.0 ± 0.1	**86.0 ± 0.1**
Caduceus (8 M)	pretrained	92.0 ± 0.5	95.0 ± 0.5	87.0 ± 0.3	84.0 ± 0.3

The best models are highlighted in bold. Best values on F1 and MCC highlighted in bold. Values are calculated on 5-fold and given in format mean ± standard deviation.

**Table 4:** LLM benchmark on G4 CUT&Tag.

Model (#params)	Type	Accuracy	AUC	F1	MCC
DNABERT (88 M)	pretrained	90.0 ± 0.5	94.0 ± 0.5	85.0 ± 0.3	73.0 ± 0.3
**DNABERT−2 (117 M)**	pretrained	97.0 ± 0.5	99.0 ± 0.5	87.0 ± 0.3	**79.0 ± 0.3**
GENA-LM (110 M)	pretrained	94.0 ± 0.5	96.0 ± 0.5	85.0 ± 0.3	76.0 ± 0.3
**HyenaDNA (7 M)**	from scratch	95.0 ± 0.5	98.0 ± 0.5	**89.0 ± 0.3**	71.0 ± 0.3
Caduceus (8 M)	pretrained	97.0 ± 0.5	99.0 ± 0.5	86.0 ± 0.3	76.0 ± 0.3

The best models are highlighted in bold. Best values on F1 and MCC highlighted in bold. Values are calculated on 5-fold and given in format mean ± standard deviation.

**Table 5:** LLM benchmark on KEx.

Model (#params)	Type	Accuracy	AUC	F1	MCC
DNABERT (88 M)	pretrained	95.0 ± 0.5	95.0 ± 0.5	80.0 ± 0.3	71.0 ± 0.3
**DNABERT−2 (117 M)**	pretrained	97.0 ± 0.5	99.0 ± 0.5	**87.0 ± 0.3**	**75.0 ± 0.3**
GENA-LM (110 M)	pretrained	94.0 ± 0.5	97.0 ± 0.5	84.0 ± 0.3	69.0 ± 0.3
HyenaDNA (7 M)	from scratch	95.0 ± 0.5	99.0 ± 0.5	75.0 ± 0.3	63.0 ± 0.3
Caduceus (8 M)	pretrained	95.0 ± 0.5	94.0 ± 0.5	62.0 ± 0.3	50.0 ± 0.3

The best models are highlighted in bold. Best values on F1 and MCC highlighted in bold. Values are calculated on 5-fold and given in format mean ± standard deviation.

The best models are highlighted in bold. Best values on F1 and MCC highlighted in bold. Values are calculated on 5-fold and given in format mean ± standard deviation.

**Fig. 1:**
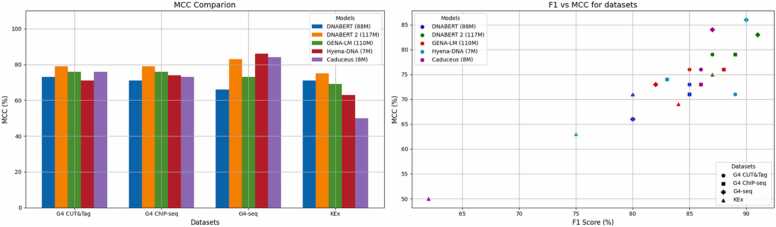
LLM model comparison. **A.** MCC comparison on four quadruplex datasets: ChIP-seq, G4-seq, G4 CUT&Tag, KEx. **B**. F1 vs MCC plot for LLM model on each of quadruplex datasets.

A graphical summary comparing LLM models based on MCC and F1 scores is presented in Fig. 1. The MCC metric for each model and each quadruplex dataset is shown in Fig. 1A. DNABERT-2 outperforms other models on the G4 CUT&Tag, G4 ChIP-seq, and KEx datasets, while HyenaDNA shows slightly better performance on the G4-seq dataset. Fig. 1B further highlights that DNABERT-2 and HyenaDNA are the top-performing models with the highest metrics for the G4-seq data set.

Benchmark on time for fine-tuning on 10 epochs to achieve best performance on balanced datasets is presented in Supplementary Table 6. Here HyenaDNA outperforms transformer-based architectures in 2–3 times and Caduceus at least in 1.3 times.

### 3.2 Interpretation of LLMs

To evaluate the performance of transformer-based LLMs, we use attention scores to rank k-mers and compare these rankings with their frequencies. As shown in Table 6, transformer-based models produce nearly identical sets of top-ranked k-mers based on attention scores. Notably, the highest attention scores do not correspond to the highest genomic frequencies. This phenomenon has been observed previously and was demonstrated in our earlier work on Z-DNA prediction using Z-DNABERT. Here, we extend these findings to quadruplexes.

**Table 6:** The top 10 6-mers ranked by attention score for each model versus the 6-mer frequency rank in the KEx experimental dataset used for tuning the model.

**Fig. 2:**
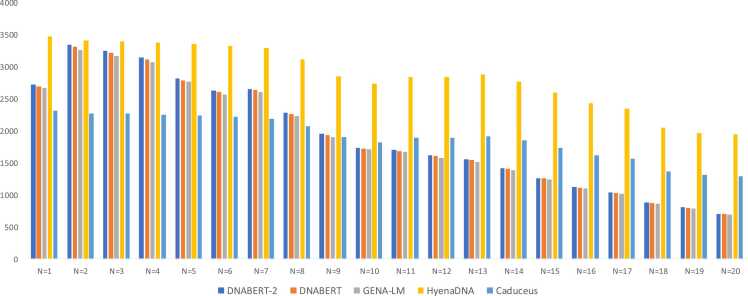
The number of patterns GGGNGGG (N = 1,20) found in LLM whole-genome predictions.

Since the transformer k-mer attentions were biased towards 1 bp loops we compared all 5 LLM whole-genome predictions for number of different loop lengths each model recovers (Fig. 2). HyenaDNA recovers more long loops GQs, three transformer-based models have similar loop length prediction pattern. Caduceus starts outperforming transformer-based models for patterns with longer loop length > 10 bp.

### 3.3 Application of Sparse Autoencoder (SAE)

**Fig. 3:**
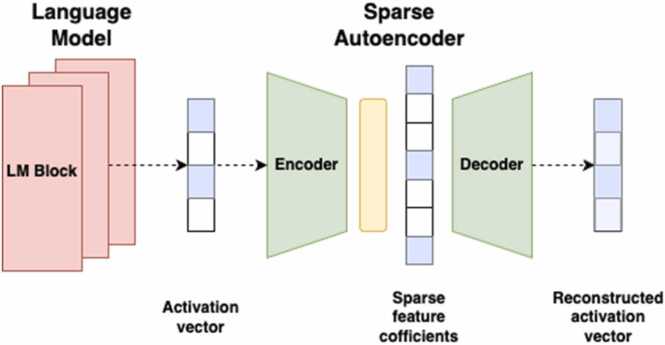
Application of sparse autoencoder to a language model.

Inspired by Engels et al. [25] and Huben et al. [26] we trained a naïve two-layer sparse autoencoder (SAE) and applied the outputs of foundation models to the SAE. Our expectation was that the dictionary elements would consist of k-mers that structure a meaningful dictionary. These elements were scored using attention scores and interpreted as features. We used our general pipeline to apply SAE to reconstruct the embeddings of a transformer-based model presented in Fig. 3. The architecture used in the Encoder-Decoder duo is different from [25] and was simplified to two dense layers. For the training of SAE we followed the method from [26] by adding KL-divergence to the training objective.

However, not all features are impactful or consistently present in G4 patterns. Engels et al. [25] proposed clustering dictionary elements by calculating Jaccard distances and then applying SAE to each cluster for reconstruction. This approach aims to reduce the feature space by eliminating non-relevant features, potentially enhancing the interpretability of the model's results. Additionally, we compared the reconstructed dictionary with the original by calculating a count score, defined as the frequency of dictionary elements. We were able to reconstruct the dictionary and assess the feature importance by extracting k-mers from a learned dictionary. Yet, our results did not provide an improvement either in model prediction or in better clustering of feature embeddings. The initial and SAE reconstructed embeddings are given in Supplementary Figures 2–5.

### 3.4 LLM performance at the genome-wide level

**Fig. 4:**
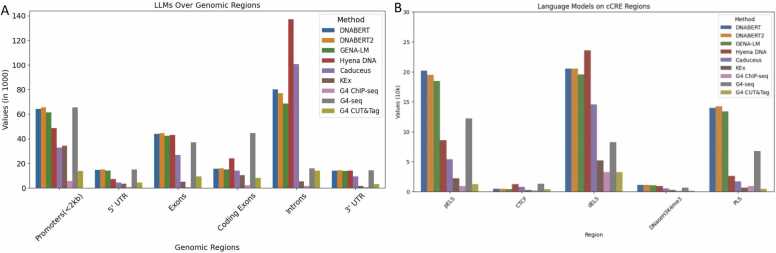
LLMs whole-genome predictions and experimental dataset distribution over (A) regulatory regions and (B) genomic regions.

To evaluate LLM model performance at the genome-wide level, we analyzed de novo quadruplexes predicted by each model. For this experiment, we selected the KEx dataset due to its more regular pattern composition and shorter sequence length of approximately 50–60 bp, which is five times narrower compared to ChIP-seq and G4-seq experimental techniques. Further, KEx was collected from living cells using a rapid chemical footprinting method. This approach avoids the problems inherent with *in vitro* based approaches where GQ may form after a cell is lysed and before the GQ detection step. A limitation of this approach is that GQ were mapped using a simple-sequence motif that is biased towards short loops and against those GQ with sequence mismatches or bulged-out unmatched bases. We generated whole-genome predictions with the five different LLM models. The distribution of the predicted quadruplexes, along with the experimental datasets across genomic and regulatory regions, is shown in Fig. 4. Additional benchmark of LLM whole-genome predictions on four experimental GQ datasets is presented in Supplementary Tables 7–10.

We can see that transformer-based models generate very similar patterns of distribution, that differ from those produced by HyenaDNA and Caduceus. We can see that HyenaDNA predicts more novel quadruplexes in introns, distal enhancers and CTCF binding cites. Caduceus has distribution pattern over regions comparable to Hyena, however the overall number of de novo predicted quadruplexes is the smallest compared to all the models.

**Fig. 5:**
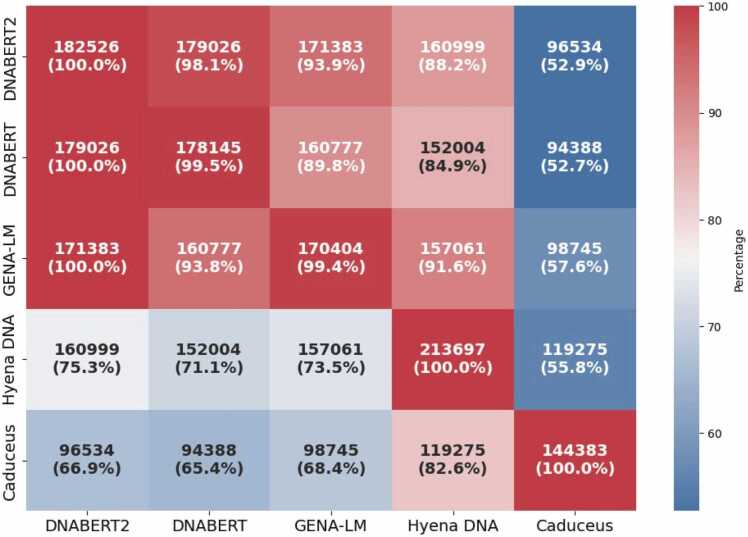
Pairwise comparison of whole-genome predictions generated by LLMs.

Intersections of whole-genome predictions generated by LLMs revealed a distant block generated by transformer-based models (Fig. 5), they all generated highly similar results. HyenaDNA has more intersections with transformer-based model than Caduceus, and Caduceus stands apart from the other models. (Fig. 5).

**Fig. 6:**
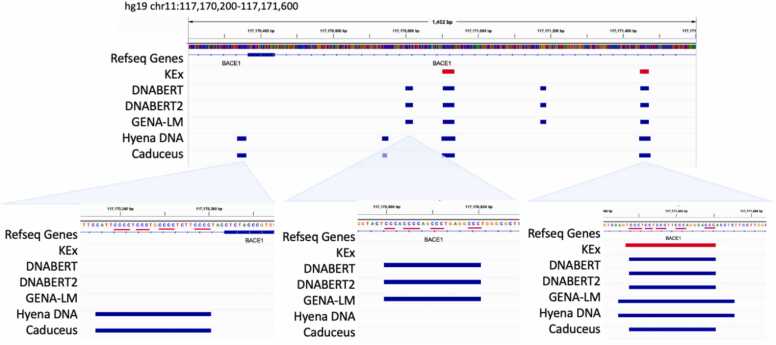
Different types of LLMs detect different quadruplexes.

An example of how different models predict different sets of quadruplexes is given in Fig. 6 for the region of BACE1 gene. At this region one can clear see that all 5 models recall well experimentally detected quadruplexes from KEx training dataset, but de novo predictions of transformer-based models differ from those of CNN-based HyenaDNA and RNN-like state space model Caduceus. Caduceus and HyenaDNA in turn predict quadruplexes that transformer-based models do not discover for some reason though detailed investigation show that all the sequences contain regular quadruplex pattern. The only explanation of why one model detects this regular pattern and ignore the other is the context, which was learned during training. These findings suggest that different types of LLM models have their own “scope of vision” depending on the size of the context and type of architecture too. At the example of quadruplexes, we see that even though all models perform almost equally on the test sets, at the whole-genome level not one model but rather an ensemble would produce the best complete map of functional genomic elements.

**Fig. 7:**
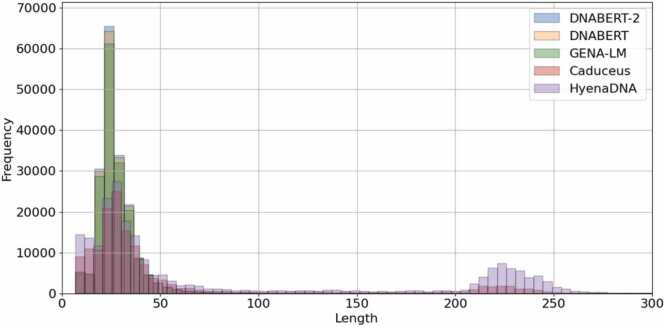
Comparison of the GQ lengths predicted by the different LLMs.

**Fig. 8:**
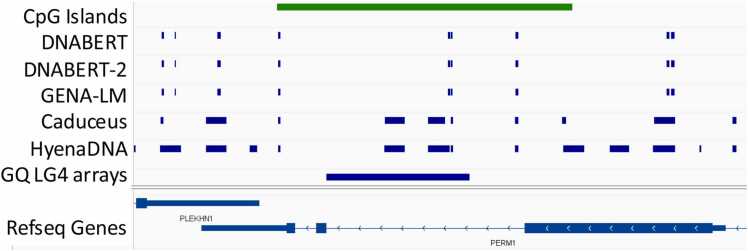
The long GQ clusters predicted by Caduceus and HyenaDNA do not always capture the full length of the GQ-LG4 arrays detected by other means [19].

One notable difference between Caduceus and HyenaDNA LLM and other models was in the length of GQ detected (Fig. 7). Inspection of these longer regions confirmed that they were detecting long clusters of GQ that are not reported by experimental detection methods, but have been previously reported as enriched in enhancer regions [19] and called LG4 (long G4). Of the 47973 GQ clusters > 200 nts predicted by Hyena, 41473 were in ENCODE enhancer regions (proximal: 6951; distal: 34522) and 9103 overlapped promoter regions. For Caduceus there were 47532 GQ clusters > 200 nts, with 10805 overlapping ENCODE enhancers (proximal: 4755; distal: 6050) and 2755 present in ENCODE promoters. However, LG4 tracks were often longer that found by HyenaDNA and Caduceus, especially in CpG rich islands (Fig. 8).

### 3.5 Embeddings clustering

**Fig. 9:**
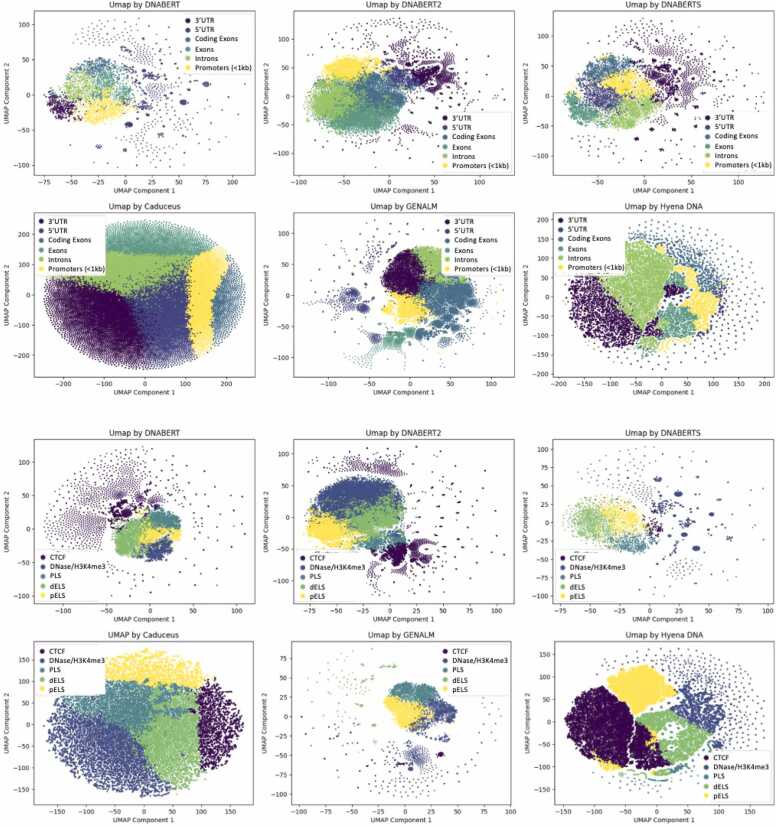
Embeddings of KEx dataset produced by DNABERT, DNABERT-2, GENA-LM, HyenaDNA and Caduceus. Visualization results are obtained by UMAP after k-means clustering.

We utilized fine-tuned models to generate embeddings for our datasets and visualized them to gain insights into the patterns and relationships captured by the models (Fig. 9). In addition to the fine-tuned models, we included DNABERT-S, a specialized model designed to produce high-quality embeddings for clustering purposes.

We applied extracted embeddings of GQs from KEx dataset and performed clustering using the UMAP algorithm, with the number of neighbors set to 30 (Fig. 7). From these UMAP representation we can see that transformer-based models have more dense and compact clustering while Hyena-DNA and Caduceus have a wider range of values for UMAP representations of embeddings reflecting the identification of GQ clusters > 200 nts. Also, we observe, as noted above, that quadruplexes from regulatory elements such as promoters (PLS), proximal enhancers (pELS) and distal enhancers (dELS) are relatively well clustered compared to quadruplexes from CTCF and HDnase/H3K4me3 regions. At the same time DNase/H3K4me3, PLS, and the enhancer-like elements (dELS, pELS) show varying degrees of overlap depending on the model, indicating that these genomic features may share more similarity in the embedding space.

CTCF regions and 3’UTR where quadruplexes are scattered all over the UMAP while at the same time being clearly separated from other groups. This result suggests that these quadruplexes are more heterogeneous than the other types, likely arising from differences in tissue-specific DNA architectures. When transformer-based models are compared to HyenaDNA and Caduceus, we see that the last two have more distinct cluster separations.

Overall, it is difficult to say that embedding from one model is better than from the other one, but they all agree between each other by clustering important regulatory and genomic regions. Additional experiments exploring the impact of varying hyperparameters, particularly the number of neighbors, are presented in the Supplementary Figures 2–5. The GQ clusters detected by HyenaDNA and Caduceus are of interest as they are not often detected by experimental datasets. One explanation is that they help nucleate the enhancer-promoter condensates that are a feature of actively transcribed regions but are not visible to approaches like CUT&Tag or chemically modified by the short time of exposure to reagents like potassium permanganate.

The Caduceus and HyenaDNA results add to of the four experimental GQ datasets examined (Supplementary Figure 10) The embeddings for three GQ datasets KEx, G4 CUT&Tag and G4 ChIP-seq overlap and are enriched in promoter regions.

## 4 Discussion and conclusions

Here we performed benchmark of five LLMs for genomics from three types of architectures – transformer-based (DNABERT, DNABERT-2, GENA-LM), CNN-based with long convolutions (HyenaDNA) and RNN-like state-space models (Caduceus) using common datasets. We evaluated each model on classification accuracy and compared their genome-wide prediction of novel functional genomic elements such as promoters and enhancers, both distal and proximal. We also assessed the overlap of these predictions with chromatin structural elements. We chose GQ to study for a number of reasons. These structures play important and varied biological roles in cells. They are encoded by genetic elements called flipons that encode information by varying their conformation under physiological conditions. Like other flipon types, GQ are subject to natural selection and show variation within a population with a potential impact on disease processes. Further, the location of tetrads that fold into GQ has been difficult to predict as the sequence context under which GQs form is quite variable. Additionally, large datasets with experimentally derived whole-genome GQ maps are available to benchmark the strengths and weaknesses of the different methods. Lastly, current genomic benchmark datasets do not annotate flipons and these genetic elements have not been previously evaluated by FMs.

Previous studies provide the basis for this work. Tang et al. [27] present a comparison of zero-shot evaluation of genomic language models versus one hot encoding representation. They found that the embeddings produced by language models in a zero-shot evaluation show overall worse performance than models trained on one-hot encoded data on a wide range of downstream tasks. However, LLMs allow fine-tuning of models to improve their performance to address this problem. With the models we tested, we found a difference in the number of epochs required for each to reach the same performance. Transformer-based models - DNABERTs and GENA–LM - perform well even in zero-shot mode and thus require few epochs for fine-tuning. In contrast, HyenaDNA and Caduceus need significant amounts of training iterations with zero-shot classification as they are not compatible with transformer-based LLMs. Their design enables the detection of features like GQ arrays that are poorly detected otherwise.

Our results show that all tested LLM models perform relatively well on all four experimental quadruplex data sets. Overall the datasets, the MCC and F1 metrics show that DNABERT-2 and Hyena-DNA outperform other models. HyenaDNA uses many fewer parameters than DNABERT-2: 7 M parameters versus 117 M 2, with our results suggesting that each method identifies different G-flipon sequence contexts.

Whole-genome predictions generated by three tested types of LLMs reflect the importance of context length and short- and long-range dependencies between functional regulatory elements. Both versions of DNABERT and GENA-LM chosen for this benchmark were pretrained using 512 bp context window while HyenaDNA and Caduceus were fine-tuned with 160 kB context. We see that HyenaDNA predicts around 3000 more quadruplexes compared to transformer-based models in distal enhancers. In genomic regions, HyenaDNA recalls considerably more intronic quadruplexes compared to other models. Caduceus is also trained using 160 kB context, however the number of whole-genome predictions is less than that of HyenaDNA. Nevertheless, HyenaDNA and Caduceus predictions group often together, suggesting they both learned a long-range dependency. Even though limited by resources, we did not employ the full capacity of those transformer-based models that utilize longer context (GENA-LM up to 36 kB, Nucleotide Transformer up to 36 kB), here we demonstrated that HyenaDNA with 16 times fewer parameters achieves comparable performance with DNABERT2 in terms of metrics. It is important to emphasize that to make a fairer comparison of transformers vs non-transformers one needs to perform experiments with a maximum available context length for each model. The current 36 kB limitation of transformer-based model provides a different set of information than the 1 Mb context evaluated by HyenaDNA and Caduseus.

With all the models tested, the LLM embeddings of quadruplexes from different genomic or regulatory regions yield distinct UMAP clusters. HyenaDNA and Caduceus clusters have more distinct boundaries while those of transformer-based models have partial overlaps. Also, the methods reveal that GQ in some regions (3’UTR or CTCF) are quite heterogeneous and do not associate into distinct clusters, consistent with the variable organization of chromatin structure in different cell types.

Previous LLM benchmark studies were performed on a set of genomic datasets (Genomic Benchmarks in [28], Genome Understanding Evaluation (GUE) in [8], BEND in [29] that include promoters, enhancers, splice-sites, gene regions, chromatin states, histone marks and transcription factor binding sites. All these benchmark datasets miss flipons, or non-B DNA structures, that were chosen for the presented study.

Our benchmark results on GQ are consistent with LLM benchmark on BEND [29] where the authors concluded that no LLMs consistently outperform the other approaches. At the same time, the study emphasized the challenge of finding enhancers that can sometimes be quite distant from the genes they regulate, making GQ dependent interactions with promoters difficult to detect without models based on a longer context. Here for the case of quadruplexes, by generating whole-genome annotations with LLMs, we demonstrated that different types of LLMs are rather complementary to each other and different models with different context length can detect different classes of functional regulatory flipons. Whether the model architecture also plays an important role in the results, and not just context length, is the subject of further research. Our study suggests that the union of maps generated by transformer-based models, long convolution-based and state space models is necessary to generate a complete map of G-flipons in the genome.

We see the direction of further research in genomic LLM interpretation, where one can understand the way foundation models learn a specific context related to the function of each genetic element. These studies will help to develop models that would be useful in finding either tissue-specific or stage-specific (for example, differentiation stage, or disease state) pathways that can be modulated therapeutically. These long GQ structures identified by Caduceus and HyenaDNA likely make important contributions to nuclear architecture and warrant more experimental analysis [19], [30].

## Funding

The study has been funded within the framework of the HSE University Basic Research Program.

## CRediT authorship contribution statement

**Cherednichenko Oleksandr:** Writing – original draft, Visualization, Validation, Methodology, Investigation, Formal analysis, Data curation. **Poptsova Maria S.:** Writing – review & editing, Writing – original draft, Visualization, Validation, Supervision, Project administration, Methodology, Investigation, Funding acquisition, Formal analysis, Data curation, Conceptualization. **Herbert Alan:** Writing – review & editing, Validation, Investigation, Formal analysis, Data curation.

## Declaration of Competing Interest

The authors declare no competing interests.

## Footnotes

Appendix A Supplementary data associated with this article can be found in the online version at doi:10.1016/j.csbj.2025.03.007.
