# Supplementary material for: Benchmarking DNA large language models on quadruplexes
Source: Comput Struct Biotechnol J. 2025 Mar 7;27:992–1000. doi: 10.1016/j.csbj.2025.03.007 (PMC11953744; doi:10.1016/j.csbj.2025.03.007)
Supplement: Supplementary file 1 — Supplementary material [file mmc1.pdf]

Supplementary Figures

A

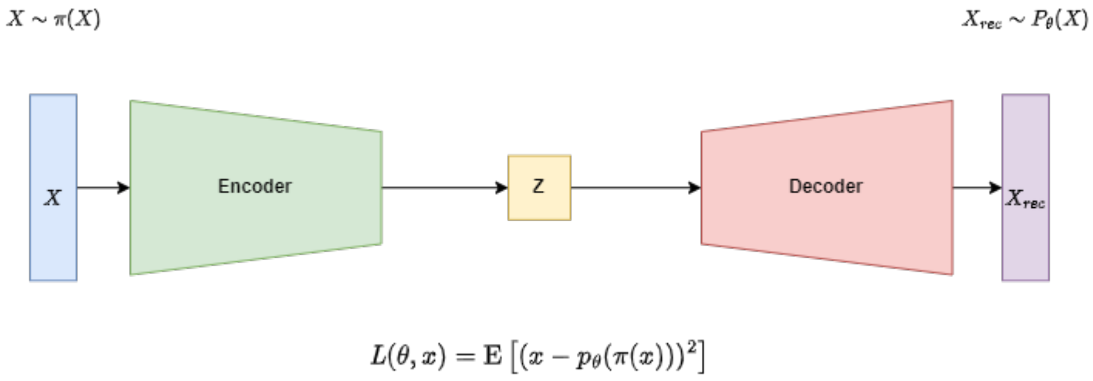

B

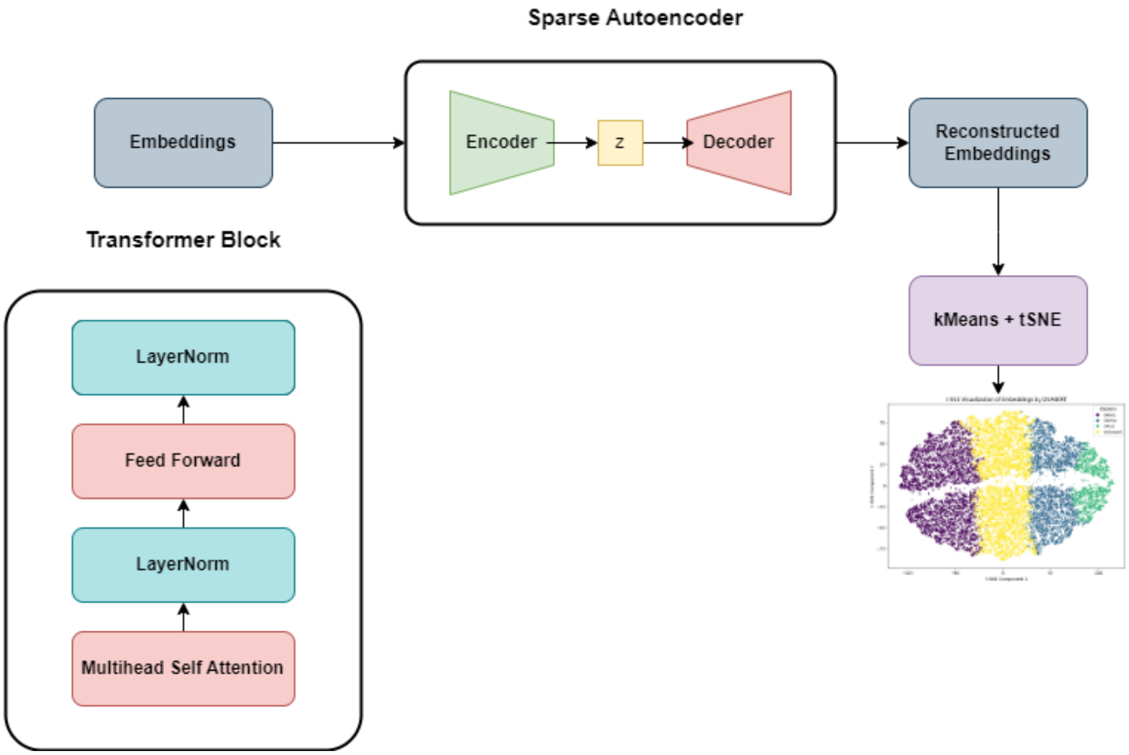

Supplementary Figure 1. A. The structure of a naive Sparse Autoencoder (SAE). The Encoder and Decoder blocks have only 2 fully connected (FC) layers. The sparsity is achieved by penalizing the activations of neurons in such a way that only a few of them can be active at any given time. For "active" neurons the activation is close to 1, while for inactive it is close to 0. It is generally accepted that most neurons should be inactive. **B.** The pipeline for application of SAE to reconstruct the embeddings of a transformer-based model. Transformer block obtains embeddings, which are then passed it to SAE. SAE reconstructs the data and it is processed by k-means clustering with tSNE visualization.

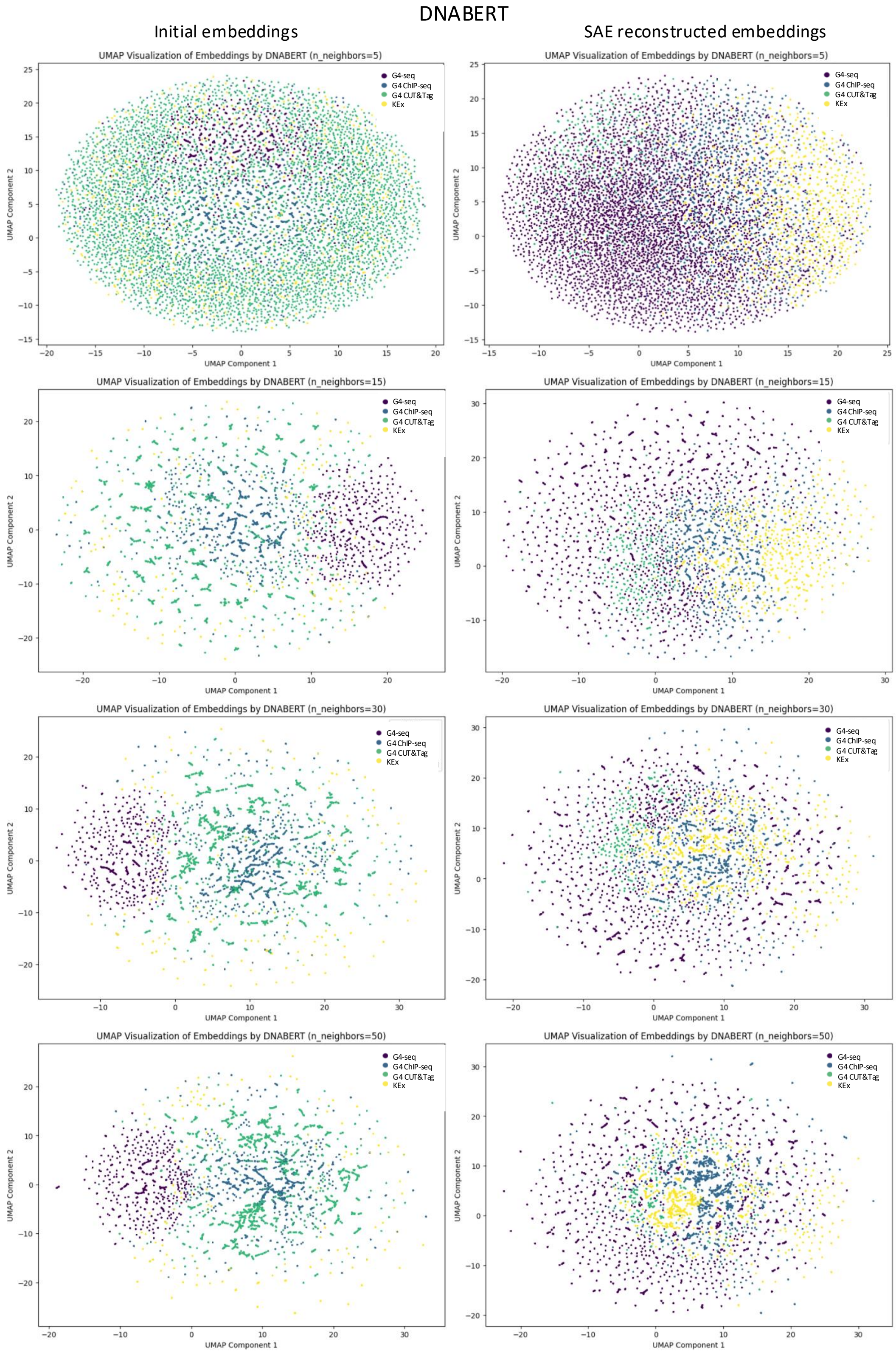

Supplementary Figure 2. DNABERT embeddings of G4 CUT&Tag, G4 ChIP-seq, G4-seq, and KEx datasets. Left column: initial embeddings. Right column: SAE reconstructed embeddings. Visualization results are obtained by UMAP after k-means; each row represents different number of neighbors.

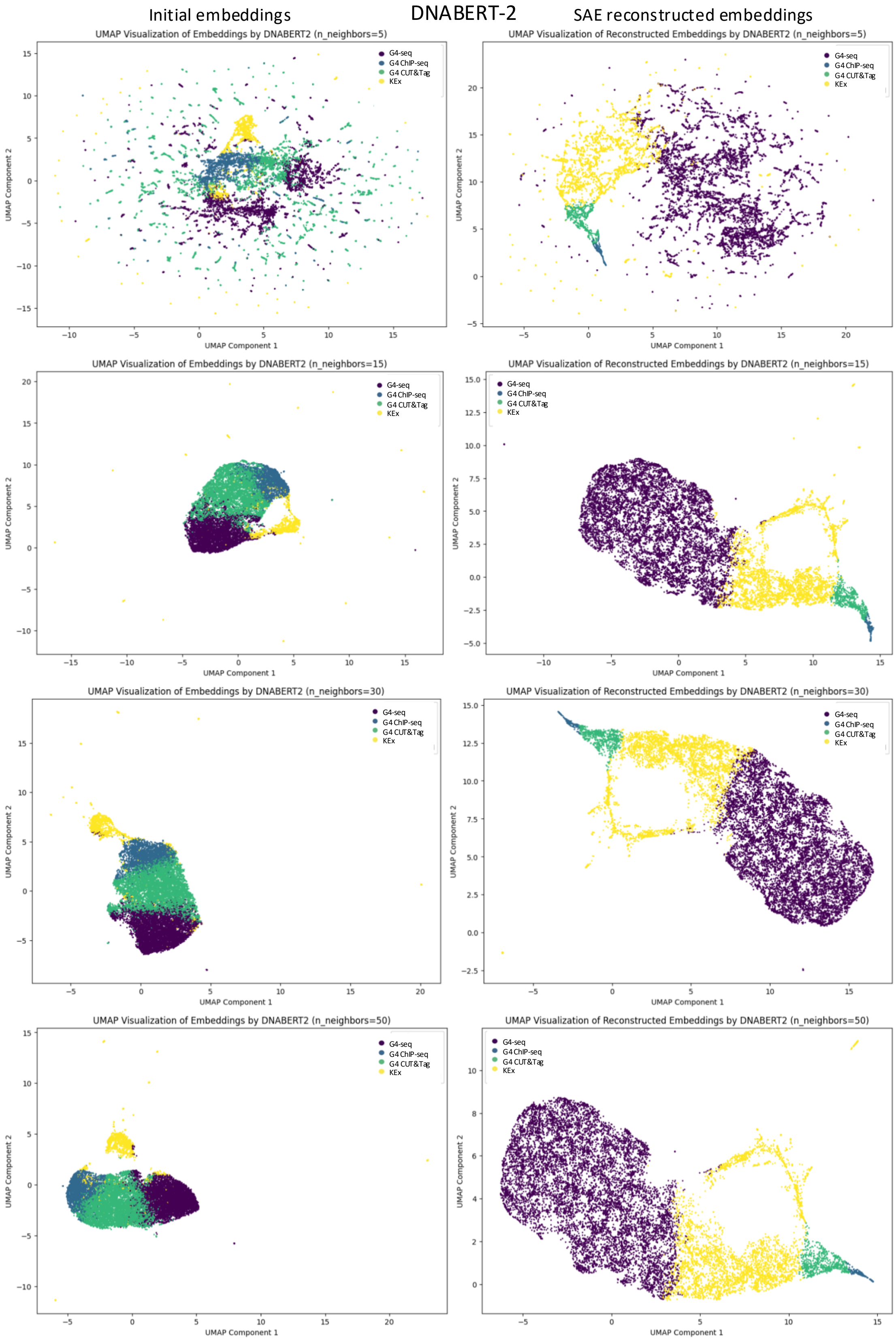

Supplementary Figure 3. DNABERT-2 embeddings of G4 CUT&Tag, G4 ChIP-seq, G4-seq, and KEx datasets. Left column: initial embeddings. Right column: SAE reconstructed embeddings. Visualization results are obtained by UMAP after k-means; each row represents different number of neighbors.

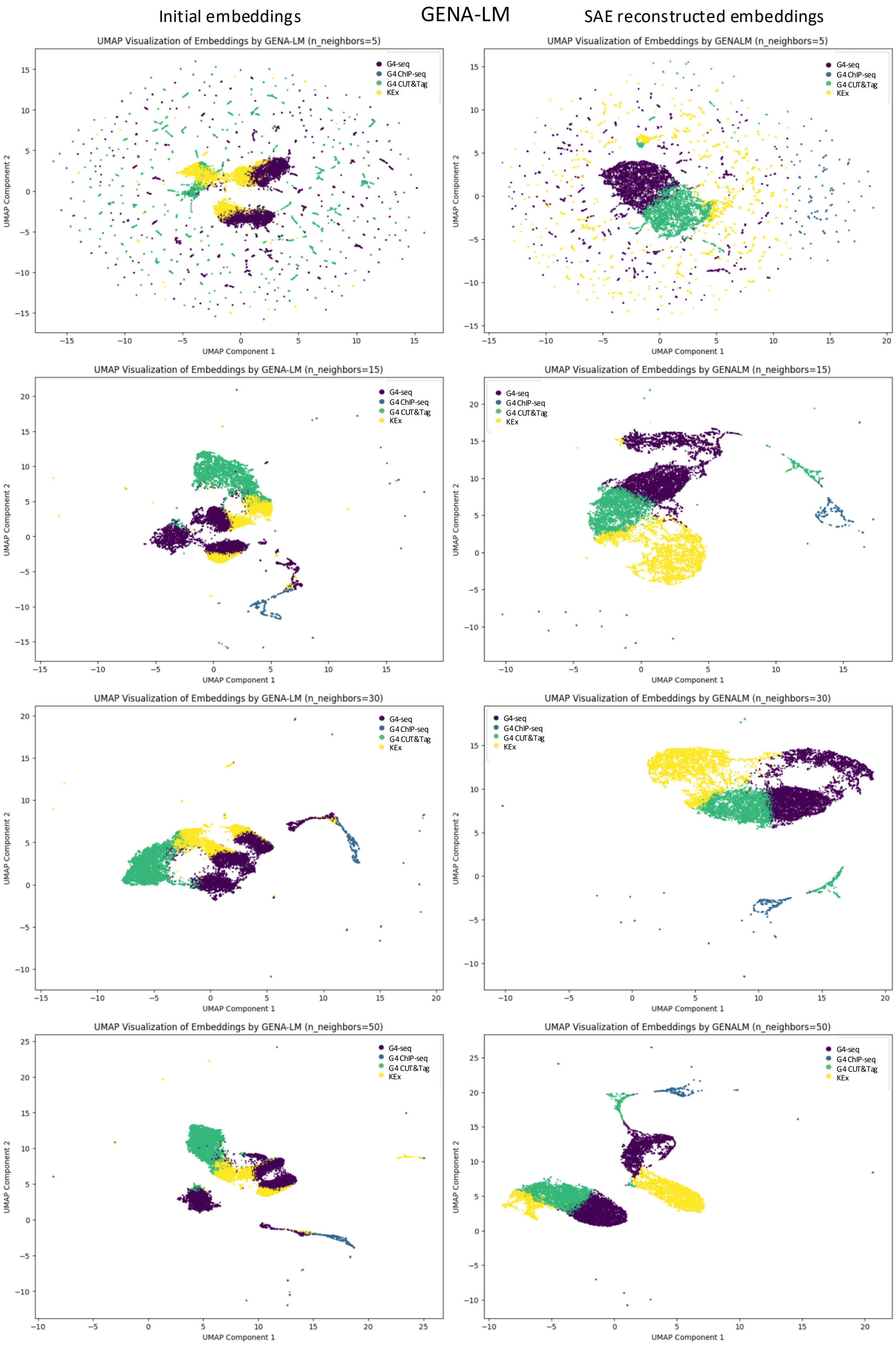

Supplementary Figure 4. GENA-LM embeddings of G4 CUT&Tag, G4 ChIP-seq, G4-seq, and KEx datasets. Left column: initial embeddings. Right column: SAE reconstructed embeddings. Visualization results are obtained by UMAP after k-means; each row represents different number of neighbors.

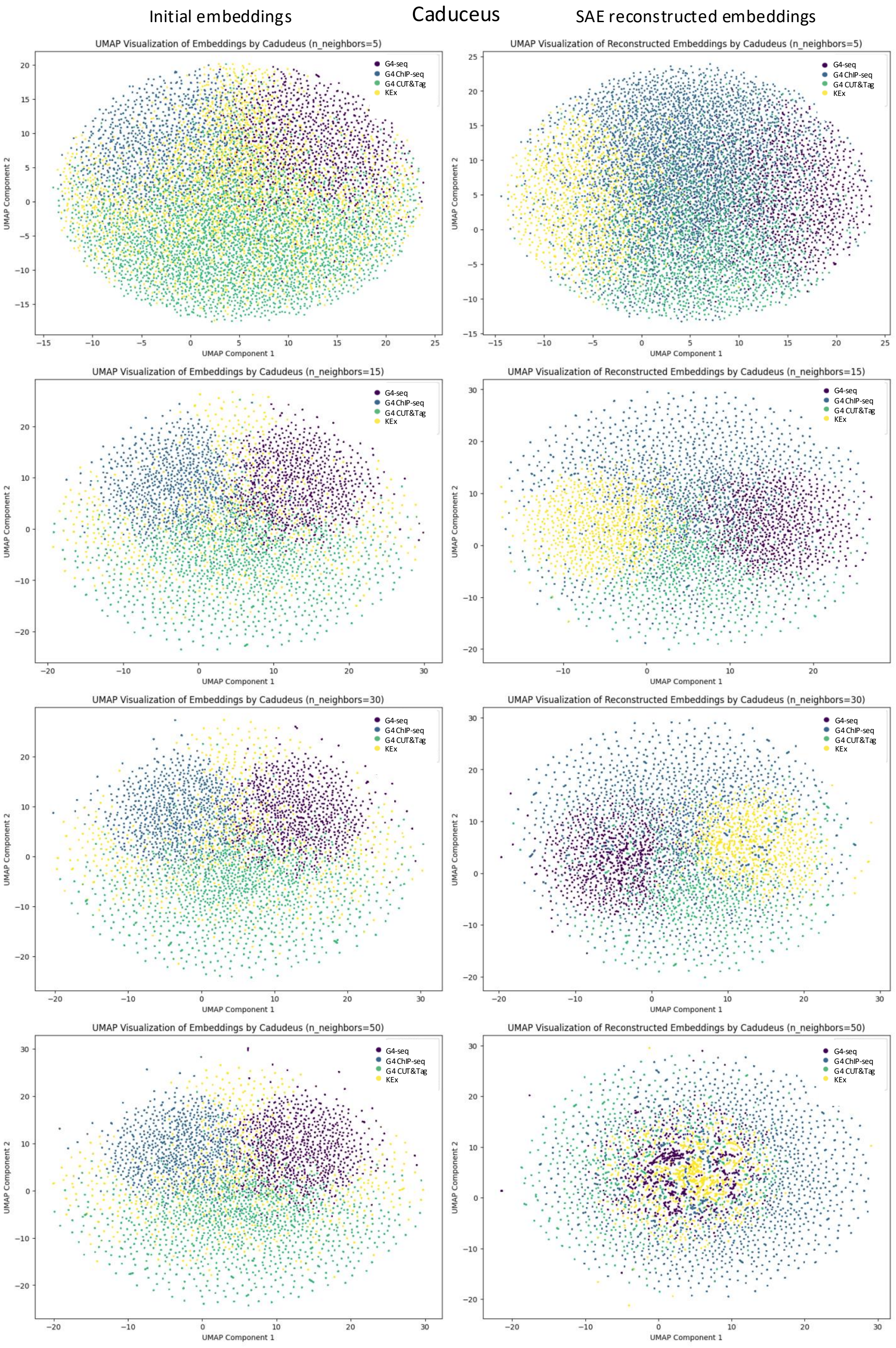

Supplementary Figure 5. Caduceus embeddings of G4 CUT&Tag, G4 ChIP-seq, G4-seq, and KEx datasets. Left column: initial embeddings. Right column: SAE reconstructed embeddings. Visualization results are obtained by UMAP after k-means; each row represents different number of neighbors.

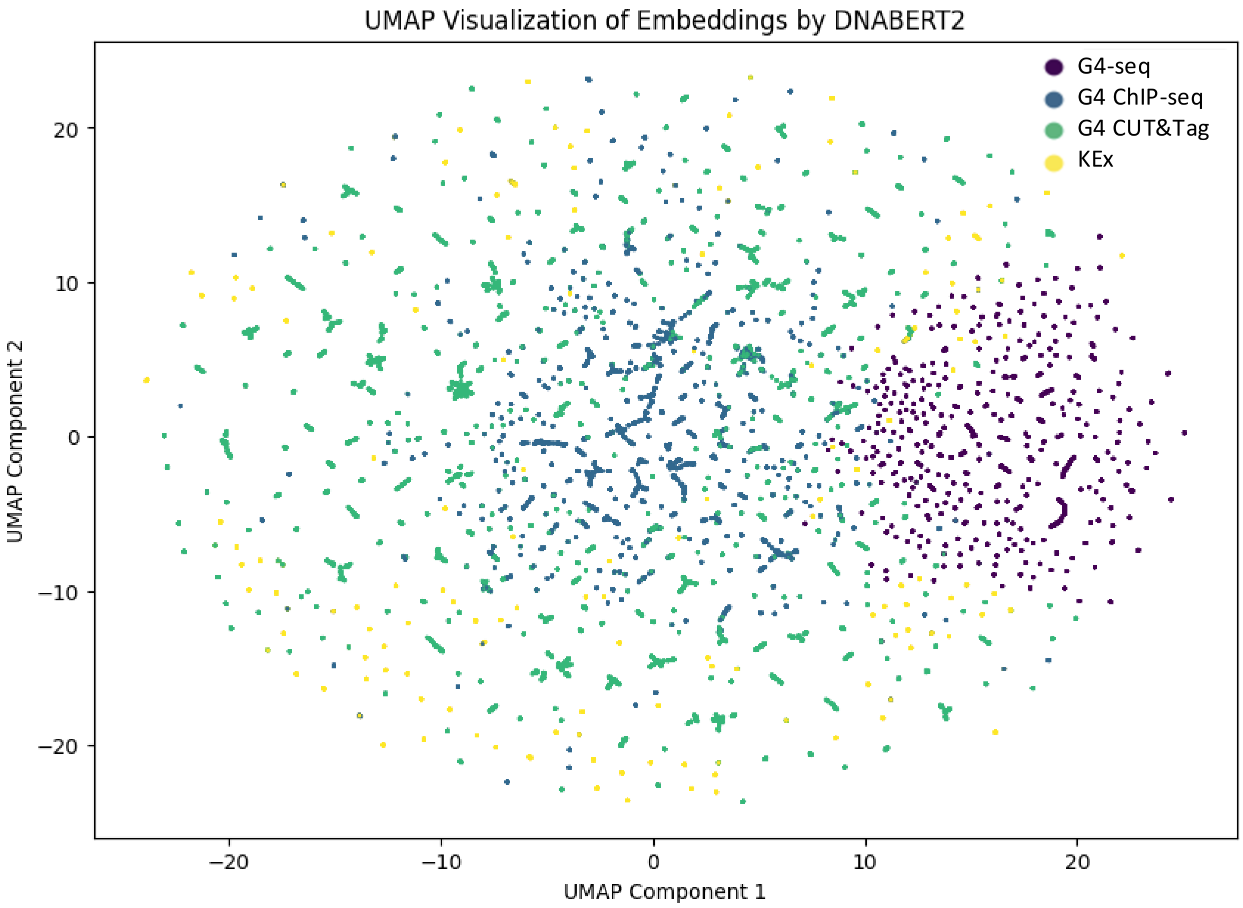

Supplementary Figure 6. The Umap plot for DNABERT-2 embeddings for four G-quadruplex datasets: G4 CUT&Tag, G4 ChIP-seq, G4-seq, and KEx.

Supplementary Tables

Supplementary Table 1. LoRA performance on G4 ChIP-seq.

Model	LoRA Params	Accuracy	AUC	F1	MCC
DNABERT	α=16, r=8, d=0.01	92 ± 7	91 ± 9	49 ± 5	27 ± 2
DNABERT 2	α=16, r=8, d=0.01	95 ± 5	93 ± 6	56 ± 2	34 ± 1
GENA-LM	α=16, r=8, d=0.01	94 ± 6	92 ± 5	50 ± 6	36 ± 2

LoRA parameters are α - is a scalable parameter, d is LoRA dropout, r is rank. The best values are highlighted in bold. Values are calculated on 5 folds and given in format mean ± standard deviation.

Supplementary Table 2. LoRA performance on G4 CUT&Tag.

Model	LoRA Params	Accuracy	AUC	F1	MCC
DNABERT	α=16, r=8, d=0.01	90 ± 5	89 ± 9	51 ± 5	37 ± 3
DNABERT 2	α=16, r=8, d=0.01	98 ± 5	95 ± 6	67 ± 2	44 ± 3
GENA-LM	α=16, r=8, d=0.01	91 ± 6	88 ± 5	50 ± 6	31 ± 3

LoRA parameters are α - is a scalable parameter, d is LoRA dropout, r is rank. The best values are highlighted in bold. Values are calculated on 5 folds and given in format mean ± standard deviation.

Supplementary Table 3. LoRA performance on G4-seq.

Model	LoRA Params	Accuracy	AUC	F1	MCC
DNABERT	α=16, r=8, d=0.01	90 ± 5	89 ± 9	51 ± 5	37 ± 3
DNABERT 2	α=16, r=8, d=0.01	98 ± 5	95 ± 6	67 ± 2	44 ± 3
GENA-LM	α=16, r=8, d=0.01	91 ± 6	88 ± 5	50 ± 6	31 ± 3

LoRA parameters are α - is a scalable parameter, d is LoRA dropout, r is rank. The best values are highlighted in bold. Values are calculated on 5 folds and given in format mean ± standard deviation.

Supplementary Table 4. LoRA performance on KEx.

Model	LoRA Params	Accuracy	AUC	F1	MCC
DNABERT	α=16, r=8, d=0.01	90 ± 5	89 ± 9	51 ± 5	37 ± 3
DNABERT 2	α=16, r=8, d=0.01	98 ± 5	95 ± 6	67 ± 2	44 ± 3
GENA-LM	α=16, r=8, d=0.01	91 ± 6	88 ± 5	50 ± 6	31 ± 3

LoRA parameters are α - is a scalable parameter, d is LoRA dropout, r is rank. The best values are highlighted in bold. Values are calculated on 5 folds and given in format mean ± standard deviation.

Supplementary Table 5. LoRA+ performance on G4-seq.

Model	LoRA Params	Accuracy	AUC	F1	MCC
DNABERT	α=16, r=8, d=0.01	91 ± 7	92 ± 7	51 ± 4	25 ± 4
DNABERT 2	α=16, r=8, d=0.01	96 ± 5	92 ± 6	59 ± 2	32 ± 3
GENA-LM	α=16, r=8, d=0.01	94 ± 6	87 ± 5	51 ± 6	35 ± 2

LoRA parameters are α - is a scalable parameter, d is LoRA dropout, r is rank. The best values are highlighted in bold. Values are calculated on 5 folds and given in format mean ± standard deviation.

Supplementary Table 6. Benchmark on time for finetuning on 10 epochs to achieve best performance on balanced datasets.

Dataset / Model	G4 CUT&Tag	G4 ChIP-seq	KEx	G4-seq
DNABERT (88M)	12h	6h	57h	31h
DNABERT-2 (117M)	16h	7h	69h	82h
GENA-LM (110M)	11h	6h	66h	82h
**HyenaDNA (7M)**	**5h**	**3h**	**14h**	**19h**
Caduceus (8M)	6h	4h	23h	31h

Supplementary Table 7. LLM Benchmark on G4 CUT&Tag on whole genome.

Model (#params)	Type	Accuracy	AUC	F1	MCC
DNABERT (88M)	pretrained	93±5	86±3	34±1	17±4
DNABERT 2 (117M)	pretrained	95±5	87±5	40±3	30±3
GENALM (110M)	pretrained	97±2	91±5	27±3	19±3
**Hyena-DNA (7M)**	from scratch	84±8	86±2	**50±3**	**33±6**
Caduceus (8M)	pretrained	95±2	99±5	33±3	16±4

The best models are highlighted in bold. Best values on F1 and MCC highlighted in bold. Values are calculated on 5-fold and given in format mean ± standard deviation.

Supplementary Table 8. LLM benchmark on G4 ChIP-seq on whole genome.

Model (#params)	Type	Accuracy	AUC	F1	MCC
DNABERT (88M)	pretrained	95±5	97±5	10±1	8±3
DNABERT 2 (117M)	pretrained	99±5	99±5	23±3	11±3
GENALM (110M)	pretrained	99±2	99±5	27±3	15±3
**Hyena-DNA (7M)**	from scratch	99±8	92±5	**32±3**	**21±3**
Caduceus (8M)	pretrained	99±2	99±5	27±3	18±3

The best models are highlighted in bold. Best values on F1 and MCC highlighted in bold. Values are calculated on 5-fold and given in format mean ± standard deviation.

Supplementary Table 9. LLM benchmark on G4-seq on full genome.

Model (#params)	Type	Accuracy	AUC	F1	MCC
DNABERT (88M)	pretrained	98±5	97±5	21±3	12±3
**DNABERT 2 (117M)**	pretrained	99±5	99±5	29±3	**19±3**
GENALM (110M)	pretrained	99±5	93±5	17±3	15±3
**Hyena-DNA (7M)**	from scratch	99±5	92±5	**36±1**	12±3
Caduceus (8M)	pretrained	91±5	83±5	10±3	8±3

The best models are highlighted in bold. Best values on F1 and MCC highlighted in bold. Values are calculated on 5-fold and given in format mean ± standard deviation.

Supplementary Table 10. LLM benchmark on KEx on full genome.

Model (#params)	Type	Accuracy	AUC	F1	MCC
DNABERT (88M)	pretrained	71±5	63±5	5±3	0±3
**DNABERT 2 (117M)**	pretrained	73±5	60±5	**15±3**	**7±3**
GENALM (110M)	pretrained	67±5	70±5	4±3	0±3
Hyena-DNA (7M)	from scratch	75±5	71±5	10±1	1±3
Caduceus (8M)	pretrained	74±5	61±5	4±3	0±3

The best models are highlighted in bold. Best values on F1 and MCC highlighted in bold. Values are calculated on 5-fold and given in format mean ± standard deviation.

Supplementary Table 11. Benchmark on time for generating whole genome predictions in hours.

Model	GPU: T4 with 16 GB	GPU: V100 with 22.5 GB	GPU: A100 with 40 GB
DNABERT (88M)	62h	49h	38h
DNABERT-2 (117M)	60h	48h	34h
GENA-LM (110M)	66h	52h	39h
**HyenaDNA (7M)**	**42h**	**34h**	**25h**
Caduceus (8M)	51h	40h	27h
